# DOES NEIGHBORHOOD DISADVANTAGE ALTER MEMORY AFTER A CANCER DIAGNOSIS? A US HEALTH AND RETIREMENT STUDY

**DOI:** 10.1093/geroni/igac059.026

**Published:** 2022-12-20

**Authors:** Ashly Westrick, Monica Ospina-Romero, Philippa Clarke, Lindsay Kobayashi

**Affiliations:** University of Michigan, Ann Arbor, Michigan, United States; University of Wisconsin, Madison, Wisconsin, United States; University of Michigan, Ann Arbor, Michigan, United States; University of Michigan, Ann Arbor, Michigan, United States

## Abstract

We aimed to determine the influence of neighborhood socioeconomic status (NSES) on long-term cancer-related memory decline of older adults. Incident cancer diagnosis and memory were assessed in the U.S. Health and Retirement Study (N=15,074, 1998-2016). Proportion of female-headed households with children, households with public assistance income, people with income below poverty, and proportion 16+ years unemployed was categorized into NSES tertiles. Linear mixed-effects models compared the standardized memory trajectories by cancer status and NSES. Cancer-free individuals living in more disadvantaged neighborhoods had worse mean memory function at age 75 and steeper memory declines than participants from less disadvantaged neighborhoods. An incident cancer diagnosis was associated with an acute memory drop at diagnosis for those living in the least disadvantaged neighborhoods. Cancer survivors had better memory prior to but not after diagnosis compared to cancer-free individuals across NSES. These findings could inform future interventions to promote cancer survivor’s long-term aging.

